# Antibody-oligonucleotide conjugate achieves CNS delivery in animal models for spinal muscular atrophy

**DOI:** 10.1172/jci.insight.154142

**Published:** 2022-12-22

**Authors:** Suzan M. Hammond, Frank Abendroth, Larissa Goli, Jessica Stoodley, Matthew Burrell, George Thom, Ian Gurrell, Nina Ahlskog, Michael J. Gait, Matthew J.A. Wood, Carl I. Webster

**Affiliations:** 1 Department of Paediatrics, John Radcliffe Hospital, and; 2 MDUK Oxford Neuromuscular Centre, University of Oxford, Oxford, United Kingdom.; 3 Medical Research Council Laboratory of Molecular Biology, Cambridge, United Kingdom.; 4 Department of Chemistry, Philipps Universität-Marburg, Marburg, Germany.; 5 Department of Physiology, Anatomy and Genetics, University of Oxford, Oxford, United Kingdom.; 6 Biologics Engineering, R&D,; 7 Discovery Sciences, R&D, and; 8 Neuroscience, Biopharmaceuticals, AstraZeneca, Cambridge, United Kingdom.

**Keywords:** Neuroscience, Gene therapy, Molecular biology, Neuromuscular disease

## Abstract

Antisense oligonucleotides (ASOs) have emerged as one of the most innovative new genetic drug modalities. However, their high molecular weight limits their bioavailability for otherwise-treatable neurological disorders. We investigated conjugation of ASOs to an antibody against the murine transferrin receptor, 8D3_130_, and evaluated it via systemic administration in mouse models of the neurodegenerative disease spinal muscular atrophy (SMA). SMA, like several other neurological and neuromuscular diseases, is treatable with single-stranded ASOs that modulate splicing of the survival motor neuron 2 (*SMN2*) gene. Administration of 8D3_130_-ASO conjugate resulted in elevated levels of bioavailability to the brain. Additionally, 8D3_130_-ASO yielded therapeutic levels of *SMN2* splicing in the central nervous system of adult human SMN2–transgenic (*hSMN2*-transgenic) mice, which resulted in extended survival of a severely affected SMA mouse model. Systemic delivery of nucleic acid therapies with brain-targeting antibodies offers powerful translational potential for future treatments of neuromuscular and neurodegenerative diseases.

## Introduction

RNA-based therapeutics are an emerging form of therapy amenable to treating multiple neurodegenerative and neuromuscular diseases, many of which are currently without a therapeutic intervention. Antisense oligonucleotides (ASOs) are synthetic, single-stranded oligonucleotides or oligonucleotide analogs designed to bind to RNAs, either messenger or noncoding RNA, using Watson-Crick base pairing. ASOs modulate the function of RNA either through steric blockage of *cis*-regulatory elements on mRNAs or by inducing RNase H1–mediated degradation of the targeted RNA (1). There are currently 8 FDA-approved single-stranded ASO drugs and many more under preclinical investigation (2). One of these, nusinersen, has been approved for the treatment of spinal muscular atrophy (SMA). SMA is a neurodegenerative disease characterized by the degeneration of lower motor neurons found within the spinal cord and by subsequent skeletal muscle atrophy. SMA is caused by a reduced level of survival motor neuron (SMN) protein due to mutations and/or deletions in the *SMN1* gene. Humans carry a redundant paralog of *SMN1*, called *SMN2*, which is unaffected in the majority of patients with SMA. However, *SMN2* has 2 main splice variants; full-length *SMN2* (*FLSMN2*) mRNA (~10%) yields functional SMN protein, while *Δ7SMN* (~90%) generates an unstable truncated SMN protein that is typically degraded (3–6). ASOs designed to bind to the intron splice suppressor N1 (ISS-N1) of *SMN2* pre-mRNA can block splicing factors from binding and thus increase the probability of exon 7 incorporation. This, in turn, increases the level of mature *FLSMN2* mRNA and functional SMN protein. The clinically successful ASO therapy for SMA (nusinersen, marketed as Spinraza) has received worldwide regulatory approval (7).

Systemic ASO therapy of neurodegenerative diseases is made challenging by the need to cross the neurovascular unit in the brain (blood-brain barrier, BBB) and spinal cord (blood–spinal cord barrier, BSCB). On their own, systemically administered ASOs are readily accumulated in the liver but are only very moderately distributed to peripheral tissues, such as skeletal muscle. Advanced chemical modifications to the backbone and sugar moieties of ASOs have improved tissue uptake but have yet to achieve significant BBB/BSCB penetration for distribution in brain and spinal cord. As such, ASO compounds, either approved (including nusinersen) or in development for neurodegenerative disease, typically circumvent the BBB/BSCB via local administration directly into the circulating cerebrospinal fluid compartment, using intrathecal delivery (8). However, intrathecal administration typically results in lower levels of ASO exposure in higher (cervical) spinal cord and cerebral regions. It is also precluded in certain SMA patients such as those with spinal column abnormalities (9). Additionally, following intrathecal administration, only low levels of ASO have been reported outside the CNS, for example in important target tissues, such as skeletal muscle and liver, both known to be affected in SMA (10). Therefore, a systemically administered ASO delivery system with biological activity in brain and spinal cord as well as in peripheral tissues is required to facilitate optimal treatment in all patients with SMA.

The transferrin receptor (TfR) is the most widely studied pathway for transport of antibody-based drugs across the BBB, BSCB, and choroid plexus (11–14). TfR is expressed on the luminal side of brain capillary endothelial cells, where it binds transferrin and traffics iron into the parenchyma. Anti-TfR antibodies and antibody fragments have successfully used this pathway to deliver drug cargoes into the brain parenchyma (11, 13, 15, 16). However, to achieve sufficient therapeutic levels of drug exposure within the brain, precision engineering of the binding affinity to TfR is required. Antibodies with a high affinity for TfR preferentially accumulate in brain capillaries but do not release efficiently to the abluminal side (17–21). Elevating the levels of antibody accumulation in the brain parenchyma requires engineering to optimize affinity, introducing pH-dependent binding and/or creating monovalent binding (21–23). The anti-mouse TfR monoclonal antibody, 8D3_130_, was designed to bind specifically to mouse TfR (mTfR) at sufficiently low affinity to permit BBB transcytosis and release it from the mTfR once exposed to the brain parenchyma (16, 24).

Our previous work has demonstrated the ability of cell-penetrating peptides to deliver phosphorodiamidate morpholino oligonucleotides (PMOs) across the BBB and BSCB at therapeutically relevant doses (25). Here we report that systemic delivery of PMOs directly conjugated to the anti-mouse TfR monoclonal antibody 8D3_130_ yields even greater bioavailability in the CNS. This induces high expression of full-length *SMN2* mRNA and SMN protein in the CNS and in peripheral tissues of an adult human SMN2–transgenic (hSMN2-transgenic) mouse model and rescues survival of severely affected neonatal SMA mice. This work provides a way forward for systemic ASO treatment in SMA and other neurodegenerative diseases treatable with ASOs, including Huntington’s disease, amyotrophic lateral sclerosis, spinocerebellar ataxia 2, and tauopathies such as Alzheimer’s disease (26).

## Results

### Synthesis and purification of anti-TfR antibody–PMO conjugates.

**Figure 1:**
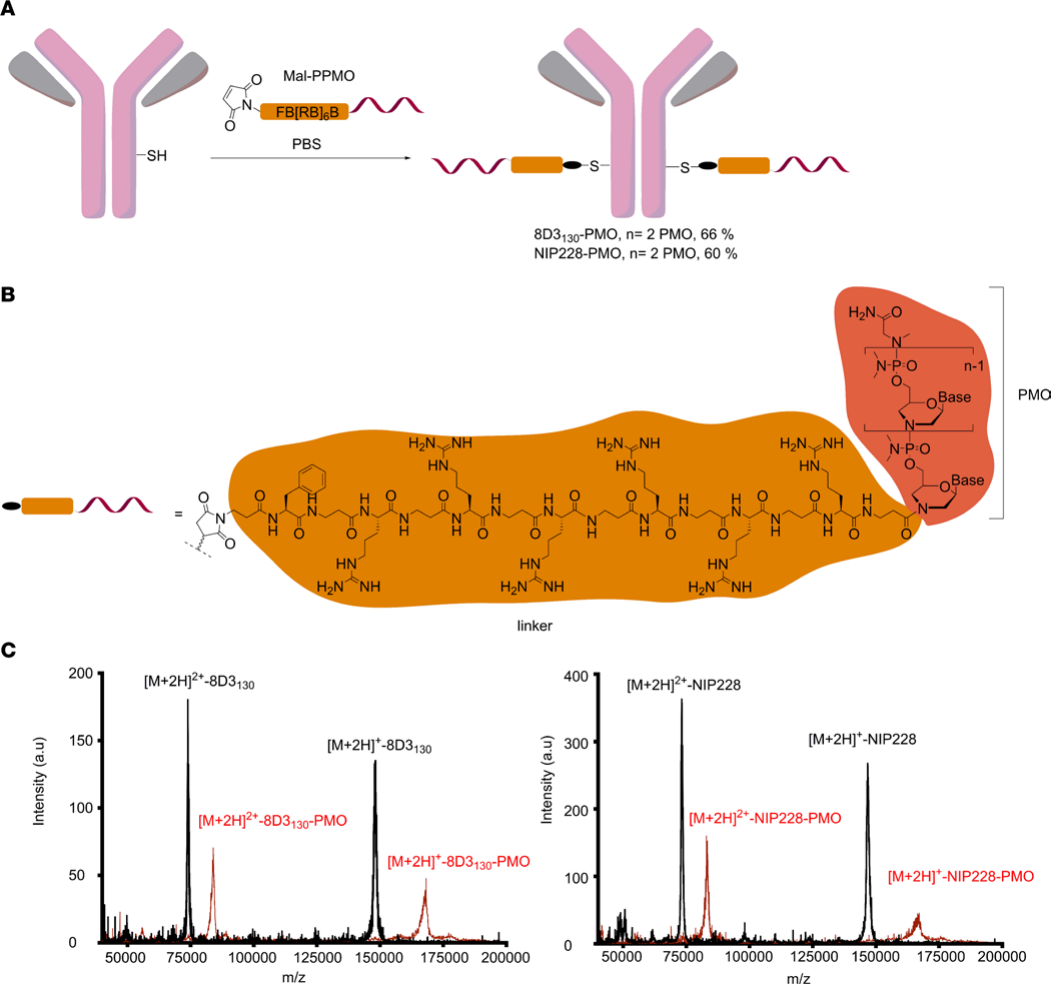
Synthesis of antibody-PMO conjugates. (**A**) Schematic of conjugation synthesis between 25-mer PMO to the free thiol group (SH) of a solvent-exposed, engineered cysteine residue in the CH2 domain of the heavy chain of either a low-affinity mouse TfR antibody (8D3_130_) or an isotype control antibody (nitrophenol, NIP228). PPMO, peptide linker-phosphorodiamidate morpholino oligonucleotide. (**B**) Chemistry of linker between PMO and antibodies. (**C**) MALDI-TOF spectra of the unmodified antibodies (black trace) and the purified antibody-PMO conjugates (red traces) for the 8D3_130_ (left) and NIP228 antibodies (right).

The ISS*-*N1 regulatory element found in intron 7 of the *hSMN2* gene is the most promising target for modulation of *SMN2* splicing. Steric masking of ISS-N1 with a fully modified ASO favors inclusion of exon 7 (27–29). We synthesized a 25-mer PMO targeting ISS-N1 and directly conjugated it to a short maleimide-functionalized peptide linker (Mal-C3-FB[RB]_6_, B = β-alanine; Figure 1A). The maleimide-functionalized PMO was then conjugated to the heavy chain of either a low-affinity anti-mouse TfR (8D3_130_) or an isotype control antibody against NIP228 (16) (Figure 1A). The PMO-antibody conjugates were purified via size-exclusion chromatography to remove any unreacted maleimide-functionalized PMO and antibody. Analysis of purified antibody conjugates by MALDI-TOF mass spectrometry revealed a fully modified product with a drug-to-antibody ratio of 2, i.e., the incorporation of 2 PMOs per antibody, which corresponded to an overall mass shift of 20.4 kDa (Figure 1, B and C, and Supplemental Figures 1 and 2; supplemental material available online with this article; https://doi.org/10.1172/jci.insight.154142DS1).

### PMO conjugation alters the pharmacokinetic properties of the 8D3_130_ antibody.

To determine the pharmacokinetic (PK) properties of the antibody-PMO conjugates 8D3_130_-PMO and NIP228-PMO, and whether these altered the plasma, brain, and spinal cord exposure over that of the antibody alone, we conducted a plasma PK, brain, and spinal cord exposure study in vivo. Adult wild-type C57BL/6J mice were administered a single intravenous (IV) dose of 20 mg/kg of 8D3_130_ (±PMO) and NIP228 (±PMO). Blood, brain, and spinal cord homogenate samples were studied at regular time intervals over a 1-week period. A MesoScale Discovery (MSD) Universal human IgG (hIgG) hIgG capture and detection assay for 8D3_130_ and NIP228 antibodies was used to determine exposure parameters.

**Figure 2:**
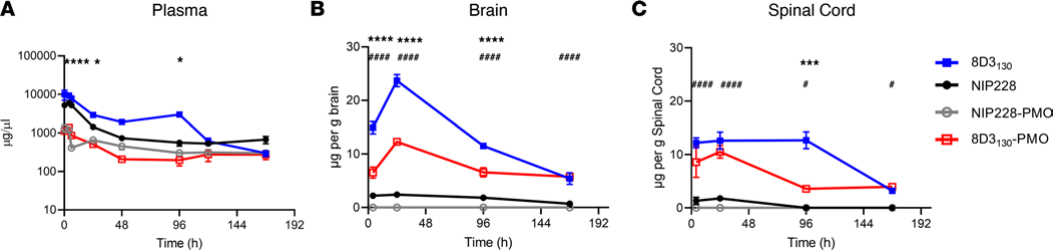
PK of antibody and antibody-PMO conjugates in mice. Plasma, brain, and spinal cord exposure following 20 mg/kg dose of 8D3_130_ (±PMO) and NIP228 (±PMO), unconjugated and conjugated to the PMO. (**A**) Plasma PK of antibodies with or without PMO over a 1-week period. High statistical significance (****) shown for 8D3_130_ 20 mg/kg versus 8D3_130_-PMO 20 mg/kg for the first 3 time points and a lower significance (*) for 24- and 96- hour time points. No statistical significance shown for NIP228-PMO 20 mg/kg versus 8D3_130_-PMO 20 mg/kg at any time point. (**B**) Brain exposure as a measure of μg compound/g brain. Statistical significance (****) shown for 8D3_130_ 20 mg/kg versus 8D3_130_-PMO 20 mg/kg for first 3 time points. Statistical significance (^####^) shown for NIP228-PMO 20 mg/kg versus 8D3_130_-PMO 20 mg/kg at all time points. (**C**) Spinal cord exposure as a measure of μg compound/g spinal cord. Statistical significance (***) shown for 8D3_130_ 20 mg/kg versus 8D3_130_-PMO 20 mg/kg at 96-hour time point. Statistical significance (^####^) shown for NIP228-PMO 20 mg/kg versus 8D3_130_-PMO 20 mg/kg at first 2 time points and a lower statistical significance (^#^) at the last 2 time points. Statistical significance (representative *P* values) for exposure in brain, spinal cord, and plasma between 8D3_130_ 20 mg/kg versus 8D3_130_-PMO 20 mg/kg (*) and NIP228-PMO 20 mg/kg versus 8D3_130_-PMO 20 mg/kg (^#^) at all time points evaluated. Statistical analysis was performed in GraphPad Prism. Data shown as the mean ± SEM, *n* = 3–4 per group. Statistical significance shown using 2-way ANOVA, where appropriate, made using Tukey test. *P, < 0.05; ***P, < 0.001; ****P, < 0.0001; ^#^*P*, < 0.05; ^####^*P*, < 0.0001.

Blood plasma was sampled at 17 minutes and 4, 6, 24, 48, 96, 120, and 168 hours postadministration. At highest, the maximum concentration (C_max_) of 8D3_130_ was 8-fold higher than its PMO-conjugated antibodies, while that of NIP228 was 4-fold higher than its control. The addition of PMO correlated negatively with clearance data, revealing a 4- and 2.5-fold higher clearance with PMO, compared with 8D3_130_ and NIP228 without PMO (Table 1). During the first 4 days postinjection, unconjugated 8D3_130_ had a significantly higher total plasma exposure (AUC_last_) than 8D3_130_-PMO (Figure 2A and Table 1). One week postinjection, the total plasma exposure (AUC_last_) was 7- and 3-fold higher for 8D3_130_ and NIP228 compared with their respective PMO-conjugated antibodies (Table 1).

Brain and spinal cord samples were collected at 4, 24, 96, and 168 hours postadministration and homogenates processed for analysis via MSD assay (Figure 2, B and C). The MSD plate-based sandwich immunoassay was formatted whereby the anti-hIgG capture antibody bound to sample hIgG (±PMO), and a specific detection antibody labeled with SULFO-TAG emitted light on electrochemical stimulation. The levels of hIgG (±PMO) in plasma, brain, and spinal cord were quantified by reference to standard curves generated using calibrator samples with a 4-parameter nonlinear regression model. At 24 hours postadministration, the C_max_ of 8D3_130_ (±PMO) in the brain (Figure 2B) was substantially 6-fold higher than NIP228 and 10-fold higher than NIP228-PMO (Table 2). At 1 week, the total exposure to the drug (AUC_last_) of 8D3_130_ and 8D3_130_-PMO was 8- and 5-fold higher, respectively, than NIP228-PMO (Table 2). Brain and spinal cord measurements for NIP228-PMO were below the lower limit of quantification for the assay for all time points and are not presented here.

In the spinal cord, C_max_ at 24 hours of 8D3_130_ (±PMO) was 6-fold higher than NIP228 and 7-fold higher than NIP228-PMO (Figure 2C and Table 1). AUC_last_ of 8D3_130_ and 8D3_130_-PMO was an impressive 51- and 22-fold higher, respectively, than for NIP228 (Table 2). Lower AUC for both 8D3_130_-PMO and NIP228-PMO are likely explained by the increased clearance of the conjugated forms.

### Dose-dependent SMN2 upregulation in brain and spinal cord by 8D3_130_-PMO.

**Figure 3:**
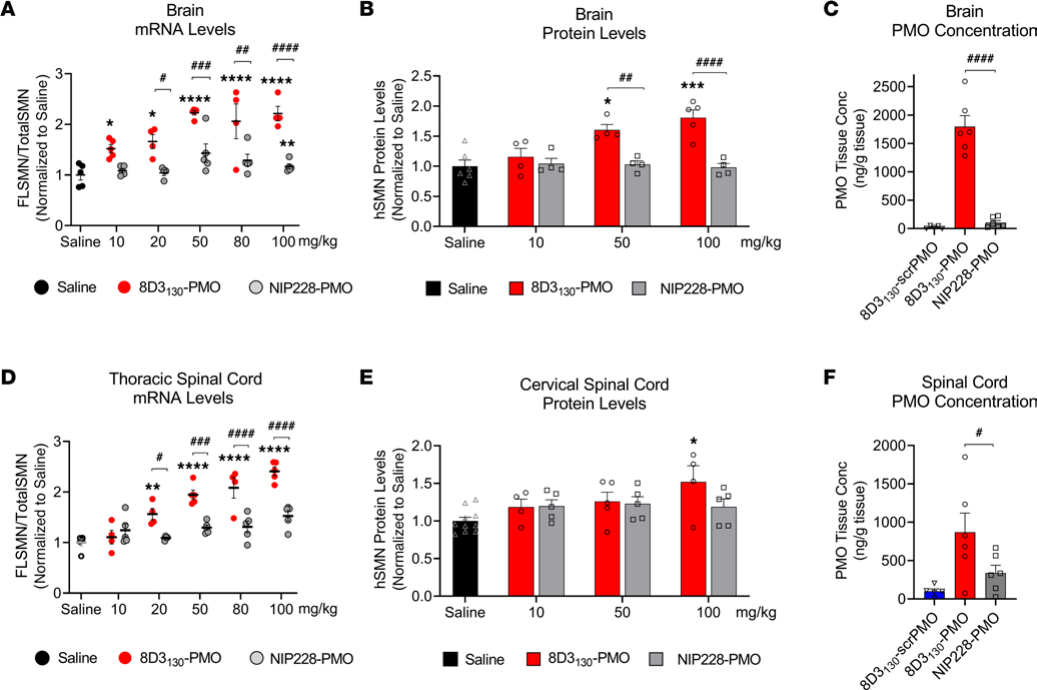
In vivo activity and concentration of antibody-PMOs in the CNS of adult transgenic mice bearing the human *SMN2* gene. Tail vein administration of 8D3_130_-PMO and NIP228-PMO was given at 8 weeks of age and tissues were harvested 7 days postadministration. Splice switching activity of the compounds compared with saline treatment on the human *SMN2* transgene was analyzed via quantitative PCR (qPCR) and Western blots (mean ± SEM). (**A**) qRT-PCR and (**B**) Western blot analysis of brain show distinct splicing activity between TfR-targeted 8D3_130_-PMO and isotype control NIP228-PMO treatment groups. (**C**) PMO concentration as determined by ELISA. 8D3_130_-scrambled PMO (8D3_130_-scrPMO) is an 8D3_130_-PMO conjugate used as negative control. (**D**) qRT-PCR from the thoracic region of the spinal cord shows elevated levels of activity. (**E**) Western blot analysis from the cervical region of spinal cord indicates little activity in this region. (**F**) PMO concentration of 8D3_130_-PMO from the whole spinal cord. Statistical significance (representative *P* values) between 8D3_130_-PMO versus saline (*) and 8D3_130_-PMO versus NIP228-PMO (^#^) was evaluated in GraphPad Prism. Data shown as the mean ± SD, *n* = 5–6 per group. qRT-PCR and Western blots were analyzed with 2-way ANOVA corrected for multiple comparisons using Dunnett’s test. ELISA for PMO concentration was analyzed with 1-way ANOVA corrected for multiple comparisons using Tukey test. *P* values adjusted to account for each comparison, confidence level 0.95%. *P, < 0.05; **P, < 0.005; ***P, < 0.0005; ****P, < 0.0001; ^#^*P*, < 0.05; ^##^*P*, < 0.005; ^###^*P*, < 0.0005; ^####^*P*, < 0.0001.

**Figure 4:**
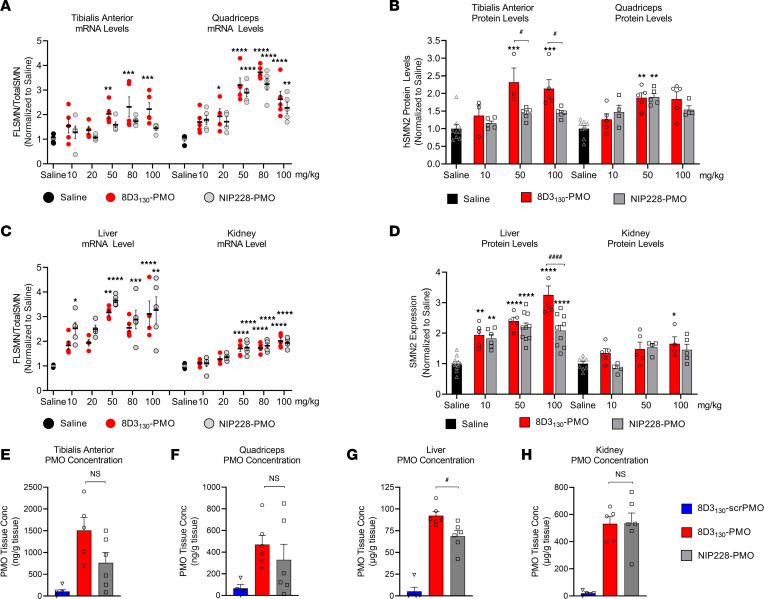
In vivo activity and concentration of antibody-PMOs in peripheral tissues of adult *SMN2*-transgenic mice. Tail vein administration of 8D3_130_-PMO and NIP228-PMO was given at 8 weeks of age and tissues were harvested 7 days postadministration. Splice switching activity of the compounds compared with saline treatment on the human *SMN2* transgene was analyzed via qPCR and Western blots (mean ± SD). qRT-PCR (**A**) and Western blot analysis (**B**) of skeletal muscles tibialis anterior (TA) and quadriceps (Quad). TA exhibited differences in activity between 8D3_130_-PMO and NIP228-PMO treatment groups. Quad show equal activity of both 8D3_130_-PMO and NIP228-PMO. qRT-PCR (**C**) and Western blot analysis (**D**) of liver and kidney tissues. Both 8D3_130_-PMO and NIP228-PMO are highly active in liver and less active in kidney. PMO concentration as determined by ELISA in (**E**) TA, (**F**) Quad, (**G**) liver, and (**H**) kidney following 50 mg/kg administration. 8D3_130_-scrPMO is used as negative control. Statistical significance (representative *P* values) between 8D3_130_-PMO versus saline (*) and 8D3_130_-PMO versus NIP228-PMO (^#^) was evaluated in GraphPad Prism. Data shown as the mean ± SD, *n* = 5–6 per group. qRT-PCR and Western blots were analyzed with 2-way ANOVA corrected for multiple comparisons using Dunnett’s test. ELISA for PMO concentration was analyzed with 1-way ANOVA corrected for multiple comparisons using Tukey test. *P* values adjusted to account for each comparison, confidence level 0.95%. *P < 0.05, **P < 0.005, ***P < 0.0005, ****P < 0.0001, ^#^*P* < 0.05; ^####^*P* < 0.0001.

The *hSMN2*-transgenic mouse strain, FVB.Cg-*Smn1^tm1Hung^* Tg(SMN2)2Hung/J, carries the entire human *SMN2* gene, lives to adulthood, and has an unaltered BBB and BSCB (30, 31). This strain is therefore an ideal adult mouse model in which to evaluate the biodistribution and biochemical efficacy of nucleic acid drug compounds that regulate *SMN2* gene expression and splicing. *hSMN2*-transgenic mice were given a single IV administration of 8D3_130_-PMO or control NIP228-PMO with a 10, 20, 50, 80, or 100 mg/kg dose. A matched volume of 5 μL/g saline administration was used for untreated controls. Each dosage group had *n* = 5 mice per group, with the exception of the 20 mg/kg dose where *n* = 4 mice. Tissues were harvested 7 days postadministration and analyzed for *SMN* mRNA splicing by quantitative reverse transcription PCR (qRT-PCR) (10, 20, 50, 80, and 100 mg/kg dosage) and SMN protein (by Western blot; 10, 50, and 100 mg/kg dosage) (Figures 3 and 4).

*FLSMN2* mRNA (qRT-PCR expression of exon 7) was normalized to total SMN (qRT-PCR expression of exons 1–2a) and plotted as a fold change over saline-treated expression (Figure 3, A and D). At the lowest dose of 10 mg/kg, *FLSMN2* expression in the brain was statistically greater than saline-treated mice (1.52 ± 0.08 versus 1.0 ± 0.098 fold change). A maximum average expression of 2.213 ± 0.145 fold expression over saline treatment was observed at 50 mg/kg dose. This activity was reflected in Western blots of SMN protein (Figure 3B). A 50 mg/kg and 100 mg/kg administration of 8D3_130_-PMO yielded 1.6 ± 0.089 and 1.8 ± 0.128 fold change in SMN protein expression over saline treatment, respectively. The high activity was also notable in the spinal cord. *FLSMN2* mRNA analysis of the thoracic region of the spinal cord yielded the highest fold change at 80 mg/kg (2.0 ± 0.2) and 100 mg/kg (2.4 ± 0.086) compared with saline-treated controls (Figure 3D). Protein was extracted from the cervical region of the spinal cord. At the 100 mg/kg dosing, SMN protein expression was significantly increased over saline treatment (1.52 ± 0.2 versus 1.0 ± 0.07), with *P* = 0.009 (Figure 3E). In contrast, NIP228-PMO–treated tissues were not found to be significantly changed from saline treatment. Discrepancies between RNA and protein results have been classically observed in both preclinical and clinical contexts of SMA in adults (9). However, the large difference between RNA and protein results in the spinal cord may also be due to morphological differences between cervical and thoracic regions of the spinal cord (32).

To determine the tissue concentration of PMO, a single IV dose of 50 mg/kg (*n* = 6 per group) was chosen given its elevated level of activity. Seven days postadministration, tissues were collected, and the concentration of PMO was determined via ELISA (33) (Figure 3, C and F). A scrambled PMO conjugated to 8D3_130_ was used to control for aberrant probe binding. The tissue concentration of PMO delivered by 8D3_130_ within the brain (1,796 ± 475 ng/g) and spinal cord (870 ± 610 ng/g) was substantially greater than PMO delivered by NIP228 (105 ± 88 ng/g and 340 ± 244 ng/g, respectively). While not directly measured, it can be assumed that active PMO accumulated in the tissue would no longer be attached to the antibody as the antibody would prevent the PMO from accessing the nucleus.

Enhanced uptake and activity were also notable in the peripheral tissues. Neuromuscular diseases typically require therapies to target skeletal muscles. SMA in particular has also been shown to affect the liver and kidney tissues (10, 34, 35). Therefore, a potential advantage of a systemically administered drug treatment would be its activity within these tissues. We analyzed the activity of 8D3_130_-PMO and NIP228-PMO in tissues from the adult SMA mice treated with a single IV administration of 10, 20, 50, 80, or 100 mg/kg dose of antibody-PMO. Two skeletal muscles of the hind limb, TA and Quad, were selected for evaluation of *FLSMN2* mRNA and protein expression over saline-treated tissues (Figure 4, A and B). Elevated levels of expression and eventual plateau starting from 50 mg/kg were notable in the TA for both mRNA and protein (Figure 4, A and B). In the Quad, a 20 mg/kg dose of 8D3_130_-PMO yielded a 2-fold enhanced expression over saline-treated Quad (1.9 ± 0.6 versus 1.0 ± 0.17 fold change, respectively). The highest level of mRNA expression was achieved at the 50 mg/kg and 80 mg/kg doses (3.2 ± 0.7 and 3.7 ± 0.3 fold change, respectively), indicating a similar plateau in efficacy at higher doses. The control NIP228-PMO also yielded elevated levels of activity in Quad but not in the TA for both mRNA (Quad: 3.2 ± 0.5 fold change and TA: 1.7 ± 0.17 fold change) and protein (Quad: 1.9 ± 0.2 fold change and TA: 1.43 ± 0.17 fold change) (Figure 4, A and B). The observed molecular effects correlated to the PMO concentration in the skeletal muscle: both 8D3_130_ and NIP228 delivered elevated levels of PMO into the TA and Quad (Figure 4, E and F). The difference in activity could be due to variations in muscle fiber composition between skeletal muscles.

Both 8D3_130_-PMO and NIP228-PMO were active in the liver and kidney (Figure 4, C and D). *FLSMN2* mRNA expression in liver was highest at 50 mg/kg for both 8D3_130_-PMO (3.17 ± 3.6 fold change) and NIP228-PMO (3.6 ± 0.2 fold change). There was no significant difference between the 2 antibody-PMOs in mRNA expression; however, the protein expression was greater in 8D3_130_-PMO treatment at 100 mg/kg (3.2 ± 0.6 fold change) than NIP228-PMO (2.1 ± 0.5 fold change), with *P* < 0.0001 (Figure 4B). Similarly, there was a significant difference between the concentration of 8D3_130_-PMO and NIP228-PMO (*P* = 0.02) in the liver (Figure 4G). In the kidney, there appeared to be a plateau in activity from 50 mg/kg both at the mRNA and at the protein levels (Figure 4, C and D). Kidney expression of *FLSMN* mRNA and protein was lower than the liver for all doses (Figure 4, C and D). While the activity of the PMO was lowest in the kidney, the PMO concentration was the highest of all tissues evaluated, indicating a nonfunctional accumulation of PMO or antibody-PMO (Figure 4H). However, the high PMO accumulation did not result in enhanced levels of kidney injury molecule-1 (KIM-1), a marker of kidney injury previously observed in animals treated with high concentrations of oligonucleotides (Supplemental Figure 3) (36).

### 8D3_130_-PMO localization throughout the brain.

**Figure 5:**
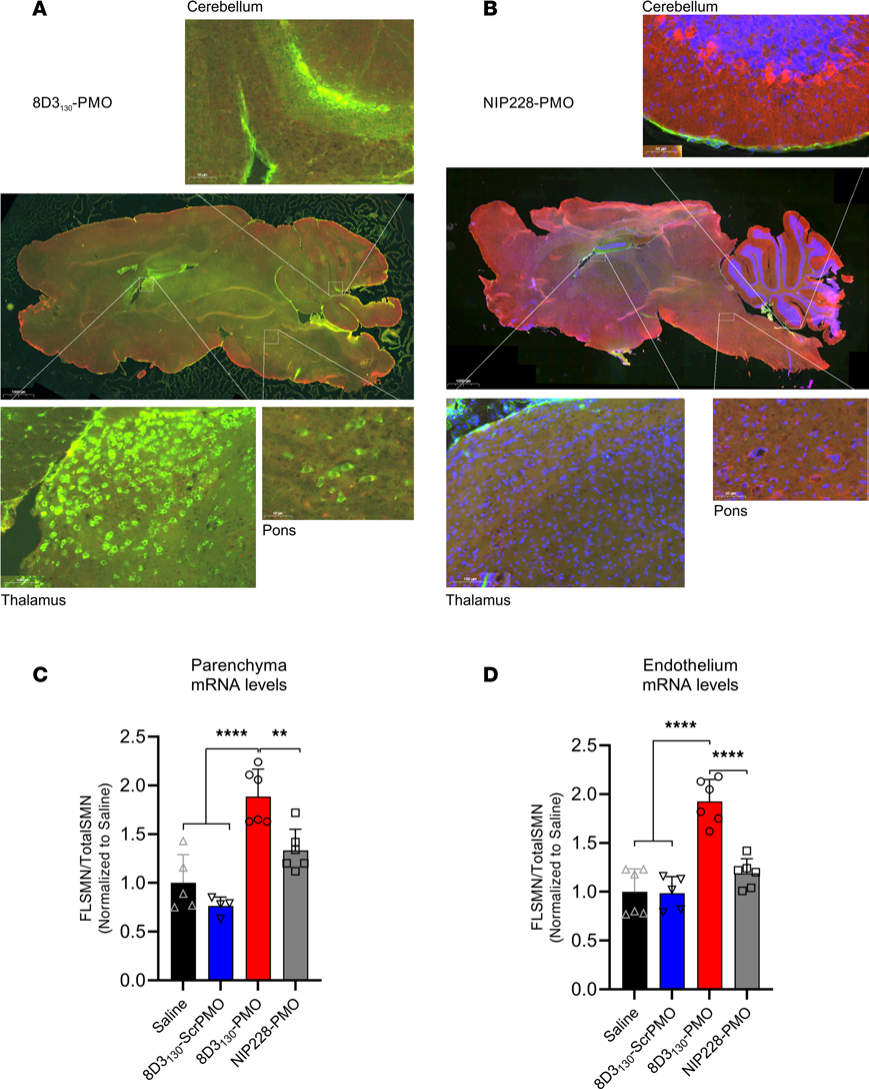
Whole-brain biodistribution of antibody-ASO conjugates. Representative images of *SMN2*-transgenic mouse brain treated with single 50 mg/kg administration of (**A**) 8D3_130_-PMO or (**B**) NIP228-PMO. The CNS was isolated from adult mice 24 hours postadministration following perfusion fixation. 8D3_130_-PMO and NIP228-PMO were identified by human secondary antibody, IgG(H+L). Whole-brain slides were imaged at original magnification 20× on 3DHistech PANNORAMIC 250 slide scanner. Images represent *n* = 3 mice. The greatest level of 8D3_130_-PMO uptake into the brain was observed in the thalamus, pons, and cerebellum regions of the brain. (**C** and **D**) *FLSMN2* expression via qPCR was analyzed in endothelium (BBB) and parenchyma of the brain fractionated by EC extraction. Mice were treated with 50 mg/kg 8D3_130_-PMO, NIP228-PMO, or 8D3_130_-scrPMO or 0.9% saline. Statistical significance (representative *P* values) was evaluated in GraphPad Prism. Data shown as the mean ± SD, *n* = 6 per group. Results analyzed with 1-way ANOVA corrected for multiple comparisons using Tukey’s test.

Previous studies have shown that anti-TfR antibodies typically require 24 hours to translocate through the endothelia of the BBB or BSCB to enter the parenchyma of the brain and spinal cord (21). To assess the topography of distribution in the brain, we dosed *hSMN2*-transgenic mice with 50 mg/kg 8D3_130_-PMO and NIP228-PMO (*n* = 3 per group) and perfused animals 24 hours later. Cryosections of the brain were immunohistochemically stained for 8D3_130_-PMO and NIP228-PMO using an Alexa Fluor 488 goat anti-hIgG secondary antibody (Figure 5, A and B). Whole-brain images revealed a fluorescent signal for 8D3_130_-PMO throughout the brain. The strongest staining of 8D3_130_-PMO was observed in the thalamus, pons, and cerebellum regions of the brain. In contrast, NIP228-PMO was not observable in the brain.

To validate that observed fluorescence correlates with drug activity within the brain parenchyma, we performed an endothelial brain tissue fractionation experiment on adult SMA mice dosed with 50 mg/kg (*n* = 6 per group) to separate the endothelium of the BBB from the brain parenchyma. Tissues were collected 7 days postadministration, and for this experiment, we included the 8D3_130_-scrambled PMO as a control. Enrichment of the endothelial cell (EC) and parenchyma fractions was validated with qRT-PCR analysis of EC markers (*Pecam1* and *Vcam1*), neuronal markers (*b-tub III* and *Map2*), as well as a glial marker (glial fibrillary acidic protein, *Gfap*) (Supplemental Figure 3). *FLSMN2* mRNA expression was measured in both fractions (Figure 5, C and D). Both saline and 8D3_130_-scrambled PMO controls yielded comparable results, indicating no activity from the 8D3_130_ antibody itself. 8D3_130_-PMO was active in both the brain parenchyma (1.89 ± 0.28 fold change) and EC (1.93 ± 0.23 fold change) fractions. NIP228-PMO activity was not significantly different from saline or 8D3_130_-scrambled PMO activity.

### 8D3_130_-PMO colocalizes to astrocytes of the spinal cord.

**Figure 6:**
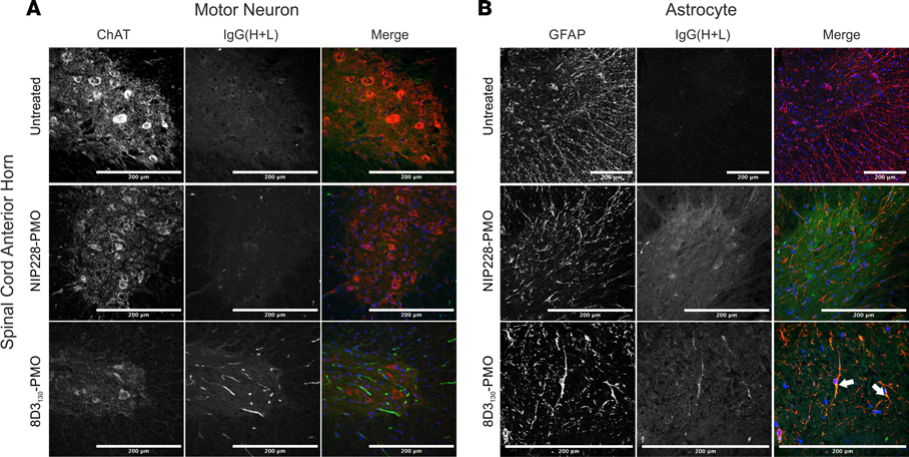
Cellular localization of 8D3_130_-PMO to the astrocytes in the spinal cord. Representative confocal images of spinal cord following single 50 mg/kg administration of 8D3_130_-PMO and NIP228-PMO. The spinal cord was isolated from adult mice 24 hours postadministration following perfusion fixation. (**A**) Motor neurons (ChAT) in the anterior horn of the spinal cord, and 8D3_130_-PMO identified by human secondary antibody, IgG(H+L), showed no overlap (merge). Fluorescence indicated a retention of the 8D3_130_-PMO [IgG(H+L)] in the vasculature. (**B**) Astrocytes of the anterior gray horn (GFAP) were colocalized with 8D3_130_-PMO [IgG(H+L)] (arrowheads). Scale bar represents 200 μm.

To evaluate which spinal cord cell populations were targeted by 8D3_130_-PMO, we dosed adult *hSMN2*-transgenic mice with 50 mg/kg 8D3_130_-PMO or NIP228-PMO and perfused animals 24 hours later. Cryosections of spinal cord were then analyzed for colocalization of 8D3_130_-PMO and NIP228-PMO to motor neurons (using choline acetyltransferase [ChAT] immunolabeling) and astrocytes (using GFAP immunolabeling). As TfR is found on the surface of neurons in the spinal cord, we expected a widespread cellular distribution of 8D3_130_-PMO (12). Instead, we observed a more cell-specific uptake of 8D3_130_-PMO in the astrocytes of the spinal cord, with minimal colocalization within motor neurons (Figure 6). The tight association of astrocytes with ECs in the BBB and BSCB regulates passage of compounds into the nervous parenchyma (37). It is therefore likely that 8D3_130_-PMO remains sequestered in astrocytes, which are the first glial population encountered in the spinal cord parenchyma.

### 8D3_130_-PMO increases FLSMN2 expression and extends survival in severely affected SMA mice.

**Figure 7:**
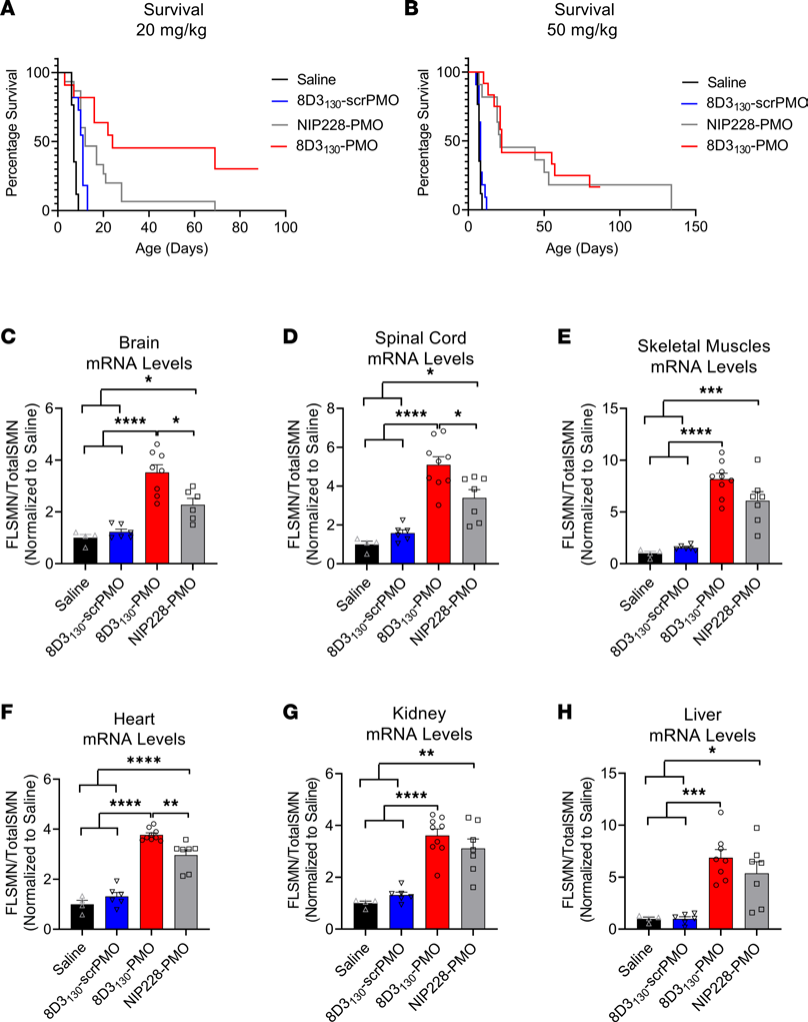
Survival and mRNA levels in severe SMA pups treated with antibody-PMOs. (**A**) Survival following single subcutaneous administration of 20 mg/kg 8D3_130_-PMO (*n* = 7), NIP228-PMO (*n* = 15), or 8D3_130_-scrambled PMO (*n* = 11) or 0.9% saline (*n* = 17). Median survival was 24, 12, 11, and 7 days, respectively. Mean survival after treatment with 8D3_130_-PMO was significantly greater than NIP228-PMO, *P* < 0.0001 (log-rank [Mantel-Cox] test). (**B**) Survival following single subcutaneous administration of 50 mg/kg 8D3_130_-PMO (*n* = 12), NIP228-PMO (*n* = 11), or 8D3_130_-scrambled PMO (*n* = 11) or 0.9% saline (*n* = 17). Median survival was 22, 21, 8, and 7 days, respectively. Both 8D3_130_-PMO and NIP228-PMO was statistically significant from 0.9% saline–treated group, *P* < 0.0001 (log-rank [Mantel-Cox] test). However, there was no statistical difference between 8D3_130_-PMO and NIP228-PMO. (**C**–**H**) qRT-PCR measure of mRNA from tissues treated with 50 mg/kg antibody-PMO and collected 7 days postadministration. Results were normalized to saline treatment controls. *FLSMN2* mRNA represented as ratio to total *SMN2* transcripts. One-way ANOVA with Tukey’s multiple-comparison test. All data represent mean values ± SD of 2 replicates. *P* value representations: ****P < 0.001, *P < 0.05.

The *hSMN2* mouse strain can be bred to produce a severe SMA phenotype in neonatal pups. SMA pups exhibit lower body weight than their unaffected littermates by day 5 and reduced movement from days 6 to 8 and subsequently die between 7 and 10 days of age (30). To assess the potential of 8D3_130_-PMO to prevent the onset of an SMA phenotype, pups were treated with a single subcutaneous administration of 20 mg/kg or 50 mg/kg dose at postnatal day 0 (PND0). Survival and tissue expression of SMN2 mRNA were evaluated (Figure 7). Median survival following single subcutaneous administration of 20 mg/kg 8D3_130_-PMO (*n* = 7), NIP228-PMO (*n* = 15), or 8D3_130_-scrambled PMO (*n* = 11) or 0.9% saline (*n* = 17) was 24, 12, 11, and 7 days, respectively. Survival of 8D3_130_-PMO treatment was statistically greater than NIP228-PMO or 8D3_130_-scrambled PMO, with *P* < 0.0001 (Figure 7A). Pups treated with the higher dose of 50 mg/kg 8D3_130_-PMO (*n* = 7) or NIP228-PMO (*n* = 8) survived a median of 22 and 21 days, respectively (*P* = NS) (Figure 7B). Only at the lower treatment dose was the difference in activity between 8D3_130_-PMO and NIP228-PMO evident. *FLSMN2* mRNA expression was measured in a separate group of pups treated at PND0 with 50 mg/kg 8D3_130_-PMO, NIP228-PMO, or 8D3_130_-scrambled PMO or 0.9% saline. Brain, spinal cord, skeletal muscles from the hind limbs, heart, kidney, and liver tissues were collected PND7. In all tissues evaluated, both 8D3_130_-PMO and NIP228-PMO significantly enhanced *FLSMN2* levels over saline-treated and 8D3_130_-scrambled PMO–treated tissues (Figure 7, C–H). 8D3_130_-PMO produced greater *FLSMN2* expression over NIP228 within brain (3.5 ± 0.84 versus 2.3 ± 0.6 fold change), spinal cord (5.1 ± 1.24 versus 3.4 ± 1.2 fold change), and heart (3.8 ± 0.24 versus 2.96 ± 0.24 fold change) (Figure 7, C, D, and F).

## Discussion

There are currently over 80 antibody drugs approved by the FDA. The majority of them treat immune-mediated diseases and various cancers, including hematologic malignancies and solid tumors. Only 9 of the 80 approved antibody drugs are antibody-drug conjugates (ADCs), all of which are approved for cancer therapies (38). These ADCs act by increasing the internalization of cytotoxic small molecules into cells expressing cancer cell membrane proteins, such as CD30 and CD33. Antibody-ASO conjugates are a newer class of drug only recently developed for therapeutic application (39).

ASO therapies have become one of the most promising forms of gene therapies for a wide range of diseases. In their naked form, ASOs are unable to pass through the BBB or BSCB and therefore require invasive modes of delivery through either direct intracerebroventricular or intrathecal administrations to treat neurodegenerative diseases. The highly successful nusinersen, an ASO targeting *SMN2* in patients with SMA, has extended survival and welfare for many children (8). However, the repeated intrathecal administrations required for treating neurodegenerative diseases subject patients to a lifetime of this invasive procedure. Reaching the CNS via systemic administration would be a major step forward in ASO therapies. We have previously used peptides to deliver ASOs to the brain and spinal cord at therapeutically relevant levels (25). Here we made use of the natural mechanisms for translocation across the BBB/BSCB by targeting the TfR with an anti-mouse TfR antibody, 8D3_130_. Systemic administration of 8D3_130_-PMO conjugates resulted in elevated levels of CNS exposure in an adult *hSMN2* mouse model while NIP228-PMO control was found to have similar effects to 8D3_130_-PMO in peripheral tissues.

TfR1 is a 97 kDa type II membrane protein expressed as a homodimer (40, 41). The TfR1 binds to iron-laden transferrin and translocates it across brain ECs. Using an anti-TfR1 antibody to translocate a cargo across the BBB has been previously successfully carried out with various cargos (11). Additionally, delivery of ASOs conjugated to anti-transferrin antibody has been used to image gene expression in rat models of brain ischemia and brain glial tumors (42–44). However, studies into antibody delivery of ASOs to the brain are limited. These early studies did not investigate the cellular biodistribution of the anti-TfR conjugates or the activity of the ASOs. The lack of in-depth imaging leaves doubts into the mechanisms of transport of the anti-TfR conjugates across the brain endothelium and of cell-specific uptake.

The anti-TfR antibody 8D3_130_ was developed from the parent antibody 8D3, which has a stronger binding affinity to the mTfR, with respective *K_D_* of 130 nM and 1.2 nM (16). 8D3 is capable of transducing across the BBB into the brain parenchyma and has been used as a fusion protein with the neuroprotective glycoprotein cytokine erythropoietin (EPO) (45). The 8D3-EPO fusion antibody garnered a modest effect in the Alzheimer’s disease mouse model (46).

8D3_130_-PMO conjugate modified the PK and activity of both the 8D3_130_ and PMO alone (Figure 2). PMO reduced the amount of 8D3_130_ assayed in the brain from the first sampling but only reduced 8D3_130_ in spinal cord 96 hours postadministration. Plasma levels of 8D3_130_ were also reduced when conjugated to PMO. This suggests that the modified antibodies are interacting with cell surfaces or extracellular matrix, thus reducing their bioavailability. However, despite the reduced plasma levels, 8D3_130_-PMO greatly improved uptake and activity of the PMO into the brain and spinal cord. To observe the effect of 8D3_130_ delivery of PMO to the brain and spinal cord, we chose an adult mouse model with 4 copies of the human *SMN2* transgene (31). This animal has no phenotype or observed disruption of the BBB or BSCB and should therefore recapitulate the biodistribution required for treating neurodegenerative and neuromuscular diseases. A 2-fold change in *FLSMN2* expression is clinically relevant for alleviating SMA disease pathology (47–49). Here, we have shown the 8D3_130_-PMO conjugate reaches above a 2-fold change to *FLSMN2* expression in the brain and spinal cord following single-dose administrations of 50 mg/kg and above. Although fold changes in protein levels are not as high, similar levels of *FLSMN2* expression in the same mouse model have only been achievable by direct brain administrations of 100–200 μg PMO (50). Therapeutically relevant levels of *FLSMN2* expression were also observable in skeletal muscles, TA, and Quad, as well as the liver, while lower levels of expression were achieved in the kidneys. In addition, doses as high as 100 mg/kg had no observable negative effects on the mice.

Despite this accumulation of compound within the kidneys, preliminary toxicity data using the above mouse model showed no significant toxicity when analyzing levels of KIM-1 (Supplemental Figure 3), a urinary biomarker of acute kidney injury (36). We hypothesize that the lack of kidney toxicity previously seen in ASO-treated mice (51) could be due to the natural path of kidney circulation for monoclonal antibodies (mAbs). The majority of mAbs are reabsorbed in proximal tubules and reenter the systemic circulation. However, the highest tissue concentration of our compounds was observed in the kidneys (Figure 4H). While the molecular mass of 8D3_130_-PMO (ca. 170 kDa) is far greater than the 60 kDa glomerular filtration cutoff, free PMO moieties resulting from metabolic cleavage (in the plasma or elsewhere) of the antibody from the PMO would be reabsorbed by the proximal tubule epithelia — which has previously been identified as the structure in the kidney with the highest localized concentrations of ASOs (52). Thus, while low levels of renal reuptake were anticipated, the antibody-PMO conjugates face the common ASO renal accumulation phenomenon described in the field (53).

The regional pattern of PMO activity and uptake into the brain and spinal cord was region and cell specific. The BBB is not homogenous throughout the CNS, and differences in permeability may allow the targeted passage into specific regions of the brain (54, 55). Immunohistochemistry of the 8D3_130_-PMO compounds indicated a particularly elevated level of uptake into pons and thalamus regions of the brain (Figure 5). A similar observation was made with another anti-TfR drug conjugate, JR-141. JR-141 is an anti-human TfR-human iduronate-2-sulfatase protein conjugate generated to treat the lysosomal storage disease mucopolysaccharidosis II (MPSII). Preclinical studies in mice and monkeys given IV administration observed JR-141 within Purkinje cells of the cerebellum and pyramidal cells in the hippocampus (13). The authors also observed widespread biodistribution of JR-141 in the heart, kidney, liver, lung, and spleen. However, they did not use an antibody isotype control, so it is unclear if this is specific for TfR binding or due to a more general antibody uptake. These results led to a phase I/II clinical trial, which demonstrated reduction in heparan sulfate (a lysosomal glycosaminoglycan inadequately catabolized in MPSII) in the cerebrospinal fluid of treated patients (56).

Investigation of spinal cord delivery showed a heterogenous patterning of 8D3_130_-PMO activity seen in *FLSMN2* and SMN protein levels across the cervical, thoracic, and lumbar regions of the spinal cord (Figure 3, D and E). This could be accounted for by the different levels of permeability between the sections of the BSCB (32). A high rate of uptake was observed in the astrocyte cell population but could not be seen in the motor neurons of the spinal cord (Figure 6). Astrocytes are a critical component of the BBB and BSCB, and they are the first point of contact for compounds translocating across the endothelium. Their expression of the Fc γ receptor, which binds to the Fc component of the immunoglobulin IgG (57), may facilitate cell uptake of the antibody-PMO conjugate. Some experimental evidence also suggests TfR expression on astrocytic cell membrane (58–60).

Astrocytes provide metabolic support to motor neurons, and low levels of SMN in astrocytes exacerbate motor neuron death in SMA (61). Increasing astrocyte-directed SMN expression extended survival and gross motor function in an SMA mouse model by rescuing defects in neuromuscular junctions and proprioceptive synapses (61, 62). Similarly, neurons cocultured with astrocytes cultured from SMA mice had reduced synaptic formation and transmission, indicating a cell-autonomous effect in SMA-derived astrocytes (63). SMN deficiency alters intracellular calcic signaling in astrocytes, which in turn may affect communication with motor neurons (63, 64). SMN deficiency in astrocytes also results in lower levels of Ephrin2 expression — an astrocytic membrane protein involved in axon guidance (63). Additionally, SMA motor neurons cocultured with SMA or wild-type astrocytes result in similar numbers of synapses and excitatory postsynaptic current, highlighting the importance of the astrocyte–motor neuron interaction in SMA (63).

Recent work has also suggested that a dysfunction of neuronal circuits is a primary causal event for SMA. In a severe SMA mouse model, the loss or reduction of proprioceptive synapses on motor neurons precedes motor neuron loss (65). Pharmacological rescue of the synapses results in improved motor behavior (66). In our study, it is possible the rescue of survival of severe SMA mice following treatment with antibody-PMOs was due to a rescue of SMN in peripheral neurons rather than the astrocytes. Further study into the 8D3_130_-PMO biodistribution of the peripheral nervous system is an exciting next step for this work.

Delivery of oligonucleotides and siRNA with antibodies has been mostly studied using the TfR as the antibody target. Early work by Penichet et al. used a system whereby an antibody was fused to avidin to act as a carrier for biotinylated pharmaceutically active drug — in their case, as a carrier for a biotinylated peptide nucleic acid oligo (67). However, only 0.12% injection dose/g was observed in brains of treated rats, far less than the 13 μg/g tissue, which equates to 2.7% injection dose/g, observed in our study. A similar compound, anti-TfR avidin linked to biotinylated luciferase-targeting siRNA, was tested in rat brain tumor expressing luciferase, and reduced luciferase levels were observed 48 hours after IV administration (68). Similar to our antibody-ASO design, Sigo et al. utilized a linker to covalently conjugate the anti-TfR (anti-CD71) to an siRNA (69). Anti-CD71–siRNA compounds were shown to be active only in peripheral tissues, liver, heart, and skeletal muscle. Unlike in our study, delivery into the CNS was not reported, and the IgG control–siRNA was not active in the skeletal muscle.

In addition to improved uptake into the CNS and peripheral tissues, 8D3_130_-PMO rescued survival in a severe mouse model of SMA. A severe SMA pup carries 2 copies of *SMN2* and deletion of exon 7 from the endogenous *mSmn*. These pups are born indistinguishable from littermates but begin to show reduction in weight and movement within a few days, with early lethality at an average of 7 days. Due to the early onset of a disease phenotype, treatment with PMO is required within a day or two after birth. The BBB and BSCB are immature at this early stage of development, leaving the brain and spinal cord exposed to high–molecular weight molecules like PMOs (25). It was unclear whether the conjugation of a PMO to 8D3_130_ would improve uptake over NIP228-PMO control given the observed activity of NIP228-PMO in peripheral tissues (Figure 4). Indeed, using a high dose of 50 mg/kg, both 8D3_130_-PMO and NIP228-PMO rescued survival and tissue expression of *FLSMN2* mRNA. However, a significant improvement in survival of 8D3_130_-PMO over NIP228-PMO was observable using a lower dose of 20 mg/kg (Figure 7), indicating the TfR is active in BBB transport at these early developmental stages and the BBB is acting as a barrier, albeit a weak one (70). Alternatively, conjugation to the anti-TfR antibody may facilitate improved cellular uptake of the PMO via TfR on the target cells compared with the control antibody.

The BBB and BSCB represent significant barriers to the delivery of biologic drugs, both protein and nucleic acid based. Improving CNS exposure using anti-TfR antibodies, which allow transcytosis across the BBB, can be exploited for the delivery of drugs to the brain. We have found that systemic dosing of an anti-TfR antibody–PMO conjugate can access the central compartment and affect the splicing of the *SMN2* mRNA to levels previously observed only with direct cerebral or intrathecal drug administration, rescuing survival in severe SMA mice. While this study provides a proof of concept for therapeutic, systemic dosing of antibody-ASO conjugates, there remains much to do in translating these results to the clinic, including understanding any toxicological liabilities. Our study offers the hope of improved therapy discovery and delivery for many debilitating neurological diseases.

## Methods

### Preparation of antibodies.

The rat anti-mouse TfR antibody 8D3 (detailed below) was mutated to reduce its affinity for mouse TfR to generate 8D3_130_ as described (16, 24). 8D3_130_ and an isotype control antibody (detailed below), used as a negative control, against the hapten NIP228 were expressed as chimeric human IgG1 molecules with a cysteine residue inserted in the CH2 domain of the heavy chain to allow site-specific conjugation of the PMOs (71). Antibodies were expressed in transiently transfected Chinese hamster ovary cells in serum-free medium as described previously (72). Cultures were maintained in a humidified incubator at 37°C, 5% CO_2_, for 14 days, after which the medium was harvested. Antibodies were purified from the medium using protein affinity chromatography followed by size-exclusion chromatography. The concentration of IgG was determined by absorbance at 280 nM using an extinction coefficient based on the amino acid sequence of the IgG (73).

### Conjugation of thiol-derivatized antibodies with Mal-C3-FB[RB]_6_-PMO.

A 25-mer maleimide-functionalized PMO conjugate targeting the ISS-N1 site of the *SMN2* gene was synthesized by conjugation of the 3′ end of the PMO to the C-terminal carboxylic acid moiety of the linker through amide coupling (Supplemental Figure 1) (74). The sequence for ISS-N1–targeted PMO (5′–3′) was GTAAGATTCACTTTCATAATGCTGG and sequence for scrambled PMO (5′–3′) was CCTCTTACCTCAGTTACAATTTATA. Both were fully modified PMOs.

IgG (15 mL at 10 mg/mL, ~1 μmol in PBS) was reduced with 40 equivalents (eq.) of Tris(2-carboxyethyl)phosphine hydrochloride (TCEP) (0.5 M in water, 80 μL, 40 μmol) for about 3 hours at room temperature under mild agitation. Samples were taken at different time points and analyzed by MALDI-TOF mass spectrometry to follow the reduction reaction. Afterward, the buffer was exchanged to Dulbecco’s PBS (DPBS) containing 1 mM EDTA by using a HiPrep 26/10 desalting column (Cytiva) at a flow rate of 10 mL/min to remove unreacted TCEP. The reduced antibody-containing fractions (~20 mL) were combined and reoxidized with 400 μL of a 50 mM (20 μmol, 20 eq.) solution of dehydroascorbic acid in DMSO for 4 hours at room temperature. Subsequently, the reaction mixture was desalted 3 times and concentrated by ultrafiltration (Ultracel 100 kDa Ultrafiltration disc with 100 mM phosphate buffer, pH 6.9, Merck Millipore) to a final volume of 12 mL.

After determination of protein concentration, a 5-fold excess of PMO-mal conjugate in 100 mM PBS buffer (pH 6.9) was added. After overnight incubation, the conjugation reaction was purified by size-exclusion chromatography (GE Healthcare, now Cytiva, HiLoad 26/600 superdex 200 pg column at a flow rate of 2 mL/min PBS). Product-containing fractions were combined and concentrated by ultrafiltration (Ultracel 100 kDa Ultrafiltration disc) to 10 mg/mL in DPBS.

### Antibody concentrations in mouse brain, spinal cord, and plasma via MSD assay platform.

Male C57BL/6 mice (Charles River Laboratories), aged 10–12 weeks, were IV injected with anti-TfR antibody (8D3_130_) or control IgG (NIP228) with or without PMO at 20 mg/kg (2 mg/mL on DPBS) or molar equivalent. IV doses were administered into a tail vein at a constant dose volume of 10 mL/kg. Antibodies were supplied in DPBS (MilliporeSigma). Following dosing, blood plasma samples were collected from the lateral tail vein (ca. 200 μL) into a Li-Hep microvette (BD Diagnostic Systems) from each of 6 animals per time point per dose group. A second sample (ca. 600 μL) was collected by cardiac puncture under isoflurane anesthesia into a Li-Hep microtainer (BD Diagnostic Systems). Following collection, blood samples were allowed to clot for 30 minutes and centrifuged at 10,000 rcf for 2 minutes at 4°C. The resultant plasma was collected and flash-frozen on dry ice for subsequent measurement of antibody concentration.

After final blood collection, the mice were perfused with DPBS at a rate of 2 mL/min for 10 minutes until the extremities appeared white. The spinal cord and brain were removed, weighed, and homogenized in 5 volumes of ice-cold PBS containing 1% NP-40 and Complete protease inhibitor cocktail tablets (Roche Diagnostics) using 2× 10 clockwise strokes with 5-second rest time. Homogenates were rotated at 4°C for 1 hour before centrifugation at 13,000*g*, 4°C, for 20 minutes. The supernatant was collected for measurement of antibody concentration. In-life phase and sample preparation were performed by Pharmaron.

Antibody concentrations in mouse plasma and brain and spinal cord homogenates were measured using the MSD assay platform. This is a plate-based sandwich immunoassay format where the anti-hIgG capture antibody binds to sample hIgG (±PMO), and subsequently, a specific detection antibody labeled with SULFO-TAG emits light on electrochemical stimulation. Levels of anti-TfR and control antibody in plasma, brain, and spinal cord samples were quantified by reference to standard curves generated using calibrator samples with a 4-parameter nonlinear regression model. Statistical analysis was performed using a 2-way ANOVA, where appropriate, made using Tukey test in GraphPad Prism. Data shown as the mean ± SEM, *n* = 3–4 per group.

### In vivo PMO activity in SMA mouse model.

The *hSMN2*-transgenic mouse [*SMN2*, FVB.Cg-Smn1^tm1Hung^Tg(SMN2)2Hung/J] was generated as previously described (30, 31) and maintained at the Biomedical Sciences Unit, University of Oxford. Handlings of Tg(SMN2)2Hung/Tg(SMN2)2Hung mice (*SMN2* offspring that carry the wild-type mouse *Smn1* gene) were conducted according to procedures authorized by the UK Home Office under the Animal (Scientific Procedures) Act 1986. In vivo dosing studies were performed in mice at 8–9 weeks of age. 8D3_130_-PMO and NIP228-PMO were diluted in 0.9% saline and given at a volume of 10 mL/kg body weight. Seven days postadministration animals were culled via rising CO_2_, and tissues were collected, flash-frozen in liquid nitrogen, and stored at –80°C until they were analyzed. Each dosage group had *n* = 5 mice per group, with the exception of 20 mg/kg dose where *n* = 4 mice. A mix of males and females was used for each study.

### RNA extraction and qRT-PCR.

RNA extraction from harvested tissues was performed using a Maxwell RSC simplyRNA Tissue Kit (Promega) and cDNA generated using ABI High Capacity cDNA Reverse Transcription Kits (Applied Biosystems, Thermo Fisher Scientific) following manufacturer’s instructions. For skeletal muscle, a 10-minute 55°C incubation of homogenized tissue was added prior to addition of lysis buffer before the RNA extraction. qRT-PCR reaction using TaqMan Fast Advanced Mastermix (Applied Biosystems, Thermo Fisher Scientific) was performed and analyzed on StepOnePlus real-time PCR system (Applied Biosystems, Thermo Fisher Scientific). *FLSMN2* and total *SMN2* transcripts were amplified using gene-specific primers (Supplemental Table 1) (75). Significance was determined via 2-way ANOVA with Dunnett’s multiple comparisons using GraphPad software (*P < 0.05, **P < 0.01, ***P < 0.001).

### Protein extraction and Western blot.

Protein was harvested from approximately 300 mg of flash-frozen tissue homogenized in RIPA buffer (25 mM Tris-HCl, 150 mM NaCl, 1% NP-40, 0.5% sodium deoxycholate, 0.1% SDS, pH 7.5) with Complete mini proteinase inhibitors (Roche). A total of 20–30 μg of protein was separated on 10% Novex Tris-Glycine gels (Invitrogen, Thermo Fisher Scientific) and transferred to PVDF membranes. Total protein stain (30% methanol, 6.7% acetic acid, 0.0005% Fast Green FCF from Merck KGaA) stain was used as a loading control and imaged prior to blocking. After blocking, human SMN protein was probed for using anti-SMN, clone SMN-KH monoclonal IgG1 (Merck Millipore, MABE230), and secondary antibody IRDye 800CW goat anti-mouse IgG (LI-COR Biosciences, 926-32210). Membranes were imaged on Odyssey FC imager and analyzed with Image Studio software (both LI-COR Biosciences).

### ELISA-based measurements of oligonucleotide concentrations in tissues.

To detect concentrations of PMOs in the tissues of treated mice, ELISAs were conducted as described in Burki et al. (33), using a phosphorothioate probe double labeled with digoxigenin (DIG) and biotin (BIO), with the following sequence (5′–3′): [DIG]--CAGCATTATGAAAGTGAATCTTAC[BIO].

### Immunohistochemistry.

*SMN2* mice (12–13 weeks old; Tg(SMN2)2Hung/Tg(SMN2)2Hung) were administered with a single IV dose of 50 mg/kg 8D3_130_-PMO or NIP228-PMO. Animals were perfused with 4% paraformaldehyde (PFA, in sterile PBS) 24 hours after treatment. Brain and spinal cords were isolated and fixed in 4% PFA overnight. Tissues were then washed 4 times in 1× PBS and cryopreserved in 30% sucrose in 1× PBS for 3 days at 4°C. Tissues were frozen in Tissue-Tek O.C.T. compound (Sakura Finetek) and stored at −80°C. Brains were cut 20 μm thick along the sagittal axis while spinal cords were sectioned transversely. Slides were stored at −80°C before proceeding. Groups of *n* = 3 were used for each treatment group.

### Whole-brain images.

Slides were thawed at room temperature (RT), rehydrated in PBS for 40 minutes, permeabilized in 0.1% Triton X (in PBS) for 10 minutes, washed twice 5 minutes in PBS, and blocked overnight at 4°C in 3% BSA (in PBS). The next day, slides were incubated with Alexa Fluor 488 goat anti-human IgG(H+L) (Invitrogen, Thermo Fisher Scientific, A-11013) at 1:500 (in 3% BSA/PBS) for 2 hours at RT. Slides were imaged at Manchester University, Bioimaging Facility, on a 3DHistech PANNORAMIC 250 slide scanner at original magnification 20×.

### IgG/Nissl NeuroTrace costaining.

Slides were thawed at RT, rehydrated in PBS for 40 minutes, permeabilized in 0.1% Triton X (in PBS) for 10 minutes, washed twice 5 minutes in PBS, and blocked overnight at 4°C in 3% BSA (in PBS). The next day, slides were incubated with Alexa Fluor 488 goat anti-human IgG(H+L) at 1:500 (in 3% BSA/PBS) for 2 hours at RT. Slides were then washed thrice 5 minutes in PBS before incubation with Nissl NeuroTrace 530/615 (Invitrogen, Thermo Fisher Scientific, N21482) at 1:200 (in 3%BSA/PBS) for 20 minutes at RT.

### IgG/ionized calcium-binding adapter molecule 1 and IgG/GFAP costaining.

Slides were thawed at RT, rehydrated in PBS for 40 minutes, permeabilized in 0.1% Triton X (in PBS) for 10 minutes, washed twice for 5 minutes in PBS, and blocked overnight at 4°C in 3% BSA (in PBS). The next day, slides were incubated with rabbit polyclonal antibody against GFAP (Abcam, ab33922) at 1:5,000 or with rabbit anti–ionized calcium-binding adapter molecule 1 antibody (FUJIFILM Wako Chemicals, 019-19741) at 1:1,000, for 24 hours in 3% BSA (in PBS) at 4°C. The next day, slides were washed thrice for 5 minutes in PBS before incubation with Alexa Fluor 594 goat anti-rabbit secondary antibody (Invitrogen, Thermo Fisher Scientific, A-11012) at 1:1,000 and Alexa Fluor 488 goat anti-human IgG(H+L) at 1:500 (Invitrogen, Thermo Fisher Scientific, A-11013) in PBS for 2 hours at RT. Samples were imaged on Olympus FV1000 confocal microscope using Fluoview software. Minimal postimaging processing was done with FIJI (ImageJ, NIH).

### IgG/ChAT costaining.

Slides were thawed at RT, rehydrated in PBS for 3 hours, and dried overnight at 4°C. The next day, slides were permeabilized and blocked by incubation for 4 hours in 0.3% Triton X + 5% BSA (in PBS), then washed for 5 minutes in PBS. Slides were incubated with a rabbit anti-ChAT antibody (EPR 16590; Abcam) at 1:250 in the blocking solution for 48 hours at 4°C. Slides were then incubated with Alexa Fluor 594 goat anti-rabbit secondary antibody at 1:750 and with Alexa Fluor 488 goat anti-human IgG(H+L) at 1:500 in PBS for 2 hours at RT.

All slides were washed 3 times for 5 minutes at RT and dried before mounting with the DAPI-containing VectaMount Permanent Mounting Medium (Vector Laboratories), then sealed with nail varnish. Slides were stored at 4°C in the dark before imaging on Olympus FV1000 confocal microscope. All images across conditions were taken on the same day for a given staining. Microscopy images were processed minimally on FIJI.

### Isolation of ECs.

Cerebral ECs from treated mice were extracted essentially as previously described (76). The brain was immediately extracted after euthanasia; cerebellum and olfactory bulbs were removed; and the remaining brain tissue was cut in half in ice-cold DMEM. The brains were individually homogenized in fresh cold DMEM. After a brief spin at 1,500 rcf and 4°C, the pelleted homogenate was resuspended in 18% dextran. The EC fraction was separated from the myelin/parenchyma layer with a 10-minute centrifugation at 5,000 rcf and 4°C. mRNA was extracted from ECs and parenchyma using Maxwell RSC simplyRNA kit according to manufacturer’s instructions. Reverse transcription and qRT-PCR were performed as before. To ascertain the quality of the EC isolation, a series of qRT-PCRs were run on the cDNA with primers (Supplemental Figure 4 and Supplemental Table 2) toward targets enriched in ECs, neurons, or glial cells.

### In vivo PMO toxicity in the SMA mouse model.

Adult hSMN2-transgenic mice were bred per regulations and treated following the method described above for the in vivo PMO activity in the SMA mouse model. Urine was collected 2 and 7 days after treatment and stored at –80°C until analyzed. KIM-1 levels in urine were analyzed using the Mouse TIM-1/KIM-1/HAVCR Quantikine ELISA Kit (R&D Systems, Bio-Techne) and normalized to urinary creatinine levels during data analysis. Urinary creatinine levels were analyzed using the clinical chemistry analyzer at MRC Harwell.

### Statistics.

Statistical analysis was performed using a 2-way ANOVA or 1-way ANOVA and corrected for multiple comparisons using Tukey test in GraphPad Prism. Detailed statistical information, including *P* value, is found within the figure legends. *P* < 0.05 was considered statistically significant.

### Study approval.

The present studies in animals were reviewed and approved by the University of Oxford Central University Research Ethics Committee. Studies were conducted according to procedures authorized by the UK Home Office under the Animal (Scientific Procedures) Act 1986.

## Author contributions

MJG, MJAW, and CIW conceptualized the project. SMH, FA, MJG, and CIW were responsible for designing the research studies. SMH, FA, MB, GT, LG, IG, and JS conducted the experiments. The original draft was written by SMH. LG, JS, NA, and MJAW were responsible for review and editing of the manuscript. SMH and FA are co–first authors in this manuscript. FA generated the antibody-oligonucleotides critical for this work. SMH was responsible for experimental design, conducting experiments, and project management; as such SMH is ordered first in the author list.

## Supplementary Material

Supplemental data

## Figures and Tables

**Table 1:**
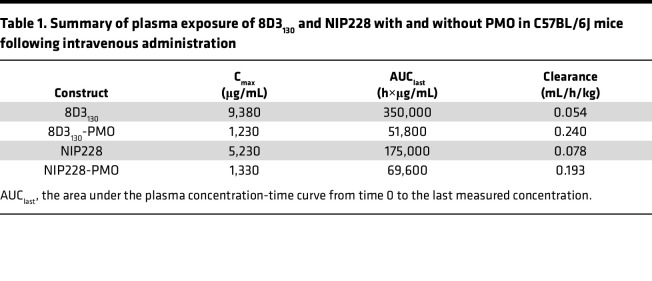
Summary of plasma exposure of 8D3_130_ and NIP228 with and without PMO in C57BL/6J mice following intravenous administration

**Table 2:**
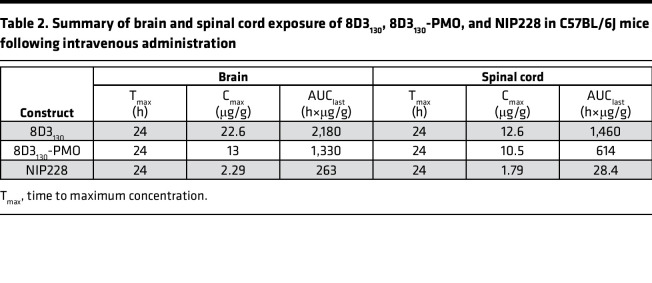
Summary of brain and spinal cord exposure of 8D3_130_, 8D3_130_-PMO, and NIP228 in C57BL/6J mice following intravenous administration

## Version 1 11/08/2022

In-Press Preview

## Version 2 12/22/2022

Electronic publication
