# Supplementary material for: Antibody-oligonucleotide conjugate achieves CNS delivery in animal models for spinal muscular atrophy
Source: JCI Insight. 2022 Dec 22;7(24):e154142. doi: 10.1172/jci.insight.154142 (PMC7614086; doi:10.1172/jci.insight.154142)
Supplement: Supplemental data [file jciinsight-7-154142-s010.pdf]

Supplementary Materials for

Antibody-oligonucleotide conjugate achieves central nervous system delivery in animal models for spinal muscular atrophy

Suzan M Hammond†, Frank Abendroth†, Larissa Goli, Jessica Stoodley, Matthew Burrell, George Thom, Ian Gurrell, Nina Ahlskog, Michael J Gait, Matthew J A Wood*, and Carl Webster*

*Corresponding author. Email: matthew.wood@paediatrics.ox.ac.uk, carl.webster@astrazeneca.com

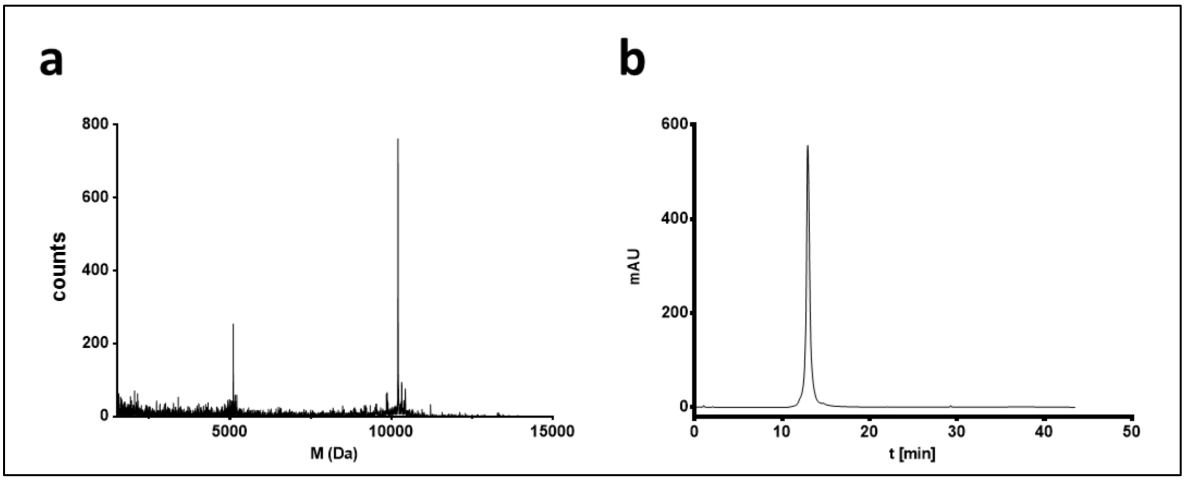

Supplemental Figure S1.

(A) MALDI-TOF spectra of 25-mer PMO targeting ISS-N1 and directly conjugated it to a short maleimide functionalized peptide linker, Mal-C3-FB[RB]$_6$-PMO. (B) LC-trace (260nm) of Mal-C3-FB[RB]$_6$-PMO.

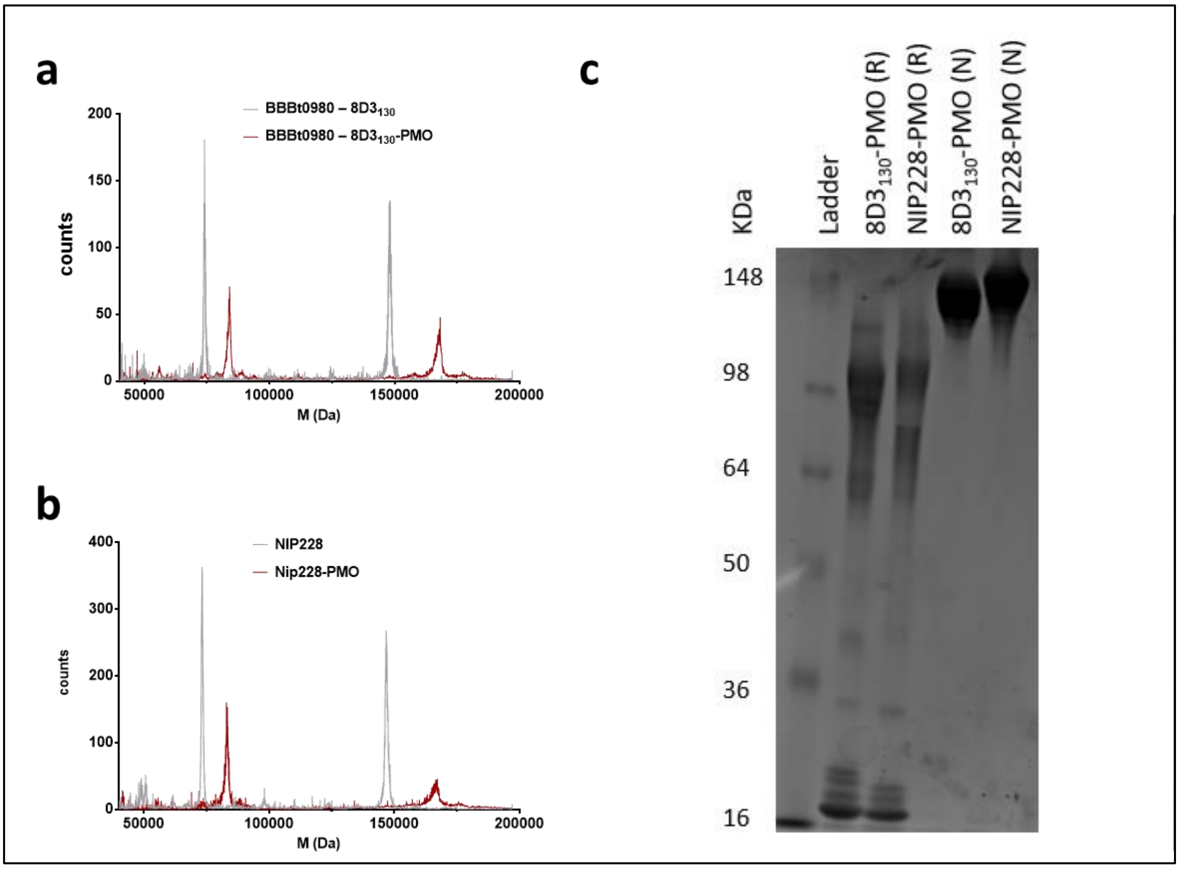

Supplemental Figure S2.

(A) MALDI-TOF spectra of $8D3_{130}$ before (grey line) and after (red line) conjugation reaction with Mal-PPMO. (B) MALDI-TOF spectra of NIP228 before (grey line) and after (red line) conjugation reaction with Mal-PPMO. (C) SDS-Page of reduced antibody-PMO conjugates (R) and non-reduced antibody-PMO conjugates (N).

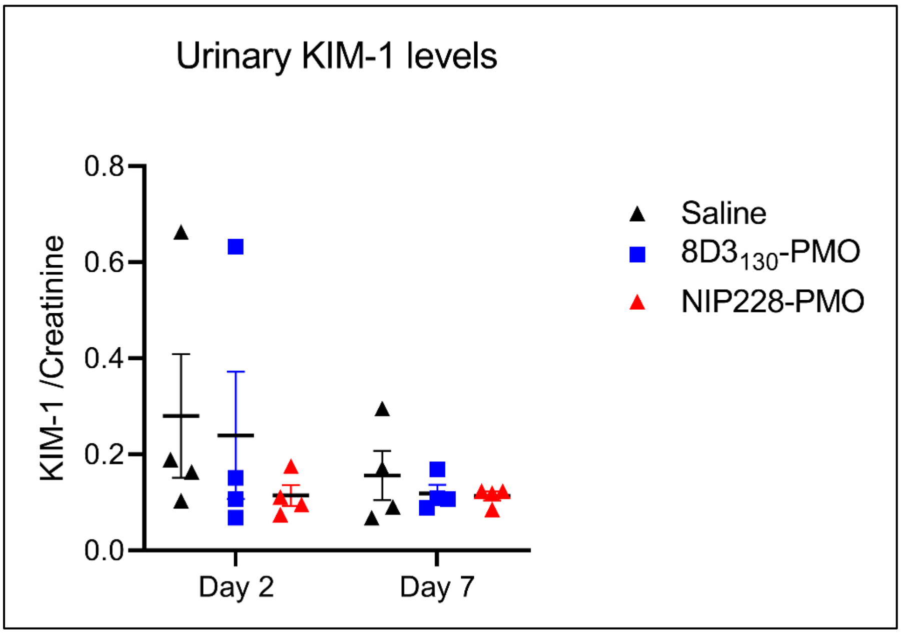

Supplemental Figure S3.
Preliminary i*n vivo* toxicity data for antibody-PMOs in male adult transgenic mice bearing the human *SMN2* gene. Tail vein administration of $8D3_{130}$-PMO and NIP228-PMO were given at 8 weeks of age and urine collected on 2- and 7-days post-administration. Urinary KIM-1 levels were measured via an ELISA and normalised to urinary creatinine levels measured using a clinical chemistry analyser (MRC Harwell Institute, UK). Data shown as mean ± S.EM., n=4 for each group. Data were analysed via 2-way ANOVA corrected for multiple comparisons using Tukey Test. P values adjusted to account for each comparison, confidence level 0.95%. *p, <0.05; **p, <0.005; ***p, <0.0005; ****p, <0.0001; #p, <0.05; ##p, <0.005; ###p, <0.0005; ####p, <0.0001; ns, not significant.

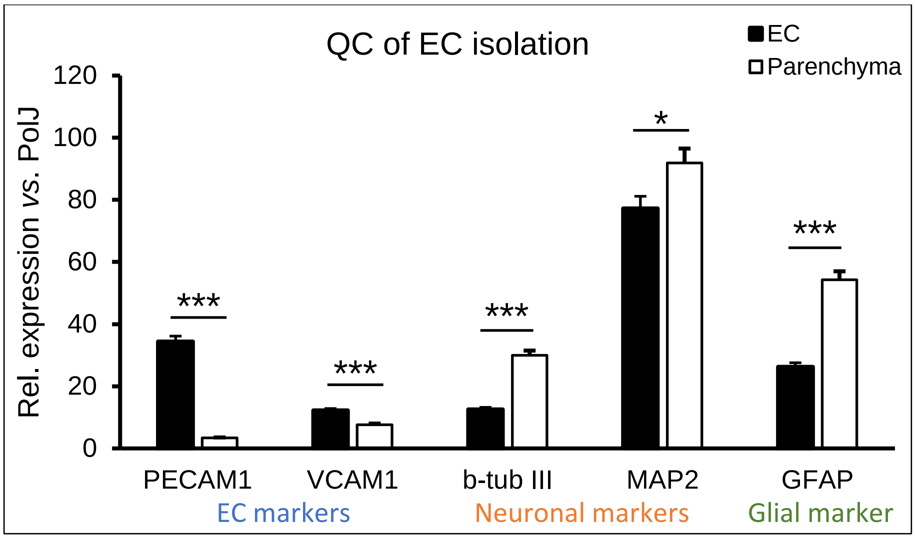

Supplemental Figure S4.

Endothelial cell isolation quality control data. The relative expression of endothelial cell (EC) markers, Pcam1 and Vcam1, were significantly higher in the EC fraction than the parenchyma fraction. On the other hand, the expression of neuronal markers β-tubulin III (Tubb3) and microtubule-associated protein 2 (Map2), was significantly higher in parenchyma fractions than in the EC. In addition, the expression of Glial fibrillary acidic protein (Gfap) was significantly higher in parenchyma fractions than in the EC (54.3±3.6 *vs.* 26.3±1.2) as expected. These all indicate that the EC isolation resulted in enriched endothelial cells.

Supplemental Table S1.

QPCR primers for SMN2 expression

Primer/Probe	FLSMN2 (5'-3')	Exon/Exon junction
Reverse Primer	TCGTTTCTTTAGTGGTGTCATTTAG	Ex8
Forward Primer	TATCATACTGGCTATTATATGGGTTTT	Ex6-Ex7
Probe	AAGGAGAAATGCTGGCATAGAGCAGC	Ex7-Ex8
	**TotalSMN2 (5'-3')**	
Reverse Primer	TCAGTGCTGTATCATCCCAAATG	Ex2a
Forward Primer	CAGGAGGATTCCGTGCTGTT	Ex1
Probe	CGGCACAGGCCAGAGCGATG	Ex1-Ex2a

Supplemental Table S2.

QPCR primers for endothelial cell isolation quality control data

Primer/Probe	***Pecam1* (NM_008816)**
Forward	TGGTTGTCATTGGAGTGGTC
Probe	CACGGGTTTCTGTTTGGCCTTGG
Reverse	TTCTCGCTGTTGGAGTTCAG
	***Vcam1* (NM_011693)**
Forward	GCAAAGGACACTGGAAAAGAG
Probe	CACTTGTGCATGGGAGACCTGTCA
Reverse	TCAAAGGGATACACATTAGGGAC
	***Tubb3* (NM_023279)**
Forward	CGCCTTTGGACACCTATTCAG
Probe	CGCCCTCCGTATAGTGCCCTTTG
Reverse	TTCTCACACTCTTTCCGCAC
	***Map2* (NM_008632.2)**
Forward	CAGGGCACCTATTCAGATACC
Probe	CAGCTCTCCGTTGATCCCGTTCT
Reverse	TCCTTCTCTTGTTCACCTTTCAG
	***Gfap* (NM_010277)**
Forward	GAAAACCGCATCACCATTCC
Probe	AGACTTTCTCCAACCTCCAGATCCGA
Reverse	CTTAATGACCTCACCATCCCG
